# The Neuroprotective Properties of the Amyloid Precursor Protein Following Traumatic Brain Injury

**DOI:** 10.14336/AD.2015.0907

**Published:** 2016-03-15

**Authors:** Stephanie Plummer, Corinna Van den Heuvel, Emma Thornton, Frances Corrigan, Roberto Cappai

**Affiliations:** 1 Adelaide Centre for Neuroscience Research, the University of Adelaide, South Australia, Australia; 2 Department of Pathology, the University of Melbourne, Victoria, Australia

**Keywords:** Amyloid precursor protein, traumatic brain injury, diffuse axonal injury, neuroprotection, heparan sulphate proteoglycans

## Abstract

Despite the significant health and economic burden that traumatic brain injury (TBI) places on society, the development of successful therapeutic agents have to date not translated into efficacious therapies in human clinical trials. Injury to the brain is ongoing after TBI, through a complex cascade of primary and secondary injury events, providing a valuable window of opportunity to help limit and prevent some of the severe consequences with a timely treatment. Of note, it has been suggested that novel treatments for TBI should be multifactorial in nature, mimicking the body’s own endogenous repair response. Whilst research has historically focused on the role of the amyloid precursor protein (APP) in the pathogenesis of Alzheimer’s disease, recent advances in trauma research have demonstrated that APP offers considerable neuroprotective properties following TBI, suggesting that APP is an ideal therapeutic candidate. Its acute upregulation following TBI has been shown to serve a beneficial role following trauma and has lead to significant advances in understanding the neuroprotective and neurotrophic functions of APP and its metabolites. Research has focused predominantly on the APP derivative sAPPα, which has consistently demonstrated neuroprotective and neurotrophic functions both *in vitro* and *in vivo* following various traumatic insults. Its neuroprotective activity has been narrowed down to a 15 amino acid sequence, and this region is linked to both heparan binding and growth-factor-like properties. It has been proposed that APP binds to heparan sulfate proteoglycans to exert its neuroprotective action. APP presents us with a novel therapeutic compound that could overcome many of the challenges that have stalled development of efficacious TBI treatments previously.

## Introduction

Traumatic brain injury (TBI) is a major public health concern, and the World Health Organisation anticipates that TBI could become the leading cause of death and disability by 2020 [1]. Despite the clear significant burden that TBI places on society, to date there are no accepted pharmacological interventions to treat TBI. Current treatment methods including osmotic agents like mannitol and hypertonic saline, or surgical interventions like decompressive craniectomy that focus primarily on stabilising the patient and managing the complications that may arise. However, these agents do not treat the underlying cause of the complications like cerebral oedema, nor are they applicable in many instances of TBI where oedema does not occur [2]. The amyloid precursor protein (APP) has a long association with TBI as its expression is dramatically upregulated in the brain following injury [3-6]. Although APP is best known and studied for its role as the source of the Amyloid-β (Aβ) peptide in the pathogenesis of Alzheimer’s disease, recent studies suggest that this upregulation of APP represents a neuroprotective response as a lack of APP impairs motor and cognitive outcomes, and enhances neuronal cell death [7]. This makes APP a promising candidate upon which to develop a therapeutic treatment for TBI subjects. As such, this review will summarise the more salient aspects of the neuroprotective properties of APP, and highlight how these are beneficial in the setting of TBI.

## Traumatic Brain Injury

TBI is a debilitating and life-threatening injury to the brain, estimated to occur in approximately 54-60 million people worldwide each year [8]. In industrialised countries, TBI causes more deaths in people under the age of 45 than any other cause [9], with the majority of cases occurring in young adults, predominantly as a result of motor vehicle accidents, followed closely by falls and assaults [10]. The consequences of TBI can be severe. Survivors are often left with lasting neurological and cognitive impairments, placing an enormous emotional, health and economic burden on society. Depression, anxiety, changes in behaviour and personality and psychiatric disorders are among the many lasting effects following TBI.

Following TBI, extensive neuronal damage is ongoing through a complex cascade of deleterious physiological events that occur in the ensuing minutes to days to weeks. Many of these deleterious events could be reversed if targeted with an appropriate therapy, preventing serious complications and reducing the burden on society.

## Pathophysiology of TBI

TBI is a complex injury that encompasses changes to both molecular and gross anatomical brain structure. Injury can occur through either impaction of the head against an object, or commonly, through acceleration/deceleration forces [11, 12]. Whilst linear acceleration movements will cause damage such as contusions and haemorrhage to superficial grey matter, rotational acceleration movements are not well tolerated by the brain, and will result in injury of greater severity [12-14]. Injury, measured on a continuum of mild, moderate and severe, will cause extensive neuronal death through a cascade of deleterious physiological events that follow the initial impact. As a result, cell death is caused by both primary and secondary injury mechanisms.

### Primary Injury

Occurring at the moment of insult, primary injury is the result of mechanical forces causing deformation of blood vessels, axons, glia and neurons [11, 14, 15], through axonal stretching, lacerations, tears, contusions and haemorrhage. Injuries are classified as either focal or diffuse, with focal injuries a result of collision forces that cause localised injuries including skull fracture and contusion. In contrast, diffuse injuries are typically the result of rapid acceleration/deceleration forces, resulting in diffuse axonal injury (DAI) [11-14]. However, both focal and diffuse injuries are often seen simultaneously [12, 16]. Unfortunately, primary injury is irreversible, and efforts should therefore focus on injury prevention, via airbags in cars and helmets for cyclists [9, 17]. In contrast, secondary injury is potentially reversible.

### Secondary Injury

Secondary injury involves a delayed and deleterious cascade of biochemical and physiological events that occur as a result of the primary injury [11, 14, 17]. Occurring in the minutes to days to weeks following the initial insult, it encompasses a range of harmful, often synergistic, effects that compound the existing injury, including glutamate excitotoxicity, oxidative stress, irreversible cell injury and death, inflammation, blood-brain-barrier (BBB) disruption, mitochondrial dysfunction and changes in ionic homeostasis [2, 9, 11, 14].

One of the most significant consequences of secondary injury following TBI is cell death, which can occur via controlled programmed cell death (PCD) through apoptosis, or unregulated death through necrosis. It has been proposed that cell death mechanisms may actually represent a continuum between apoptotic and necrotic pathways [18]. It is well established that free radicals, increases in intracellular calcium and excitatory amino acids are all implicated in the development of apoptosis. However, it is now also believed that a shift in the balance between pro-apoptotic factors like Bcl-2, Bcl-x and extracellular signal-regulated kinases, and anti-apoptotic factors, such as Bax, c-Jun N-terminal kinase, tumor-suppressor gene, p53 and calpain and caspase proteases, also play a role in influencing cell death following trauma [18].

Many of these serious secondary injury events have the potential to be reversed [9], but without treatment will often compound leading to further consequences such as ischaemia, brain dysfunction, cerebral oedema and often death [17]. Fortunately, the delayed onset and potentially reversible nature of these secondary events provides a novel window of opportunity for a therapy to reduce neuronal damage and help limit/prevent the associated morbidity and mortality [9]. It has been suggested that therapeutic interventions should be multifactorial in nature, targeting multiple elements of the secondary injury cascade [9, 19, 20]. One proposed approach is to emulate the body’s endogenous repair response.

### Diffuse Axonal Injury

One of the most common and significant features of TBI is DAI, which currently lacks an efficacious treatment. DAI is defined as the occurrence of diffuse damage to axons in the cerebral hemispheres, in the corpus callosum, in the brain stem and sometimes in the cerebellum resulting from a head injury [21]. DAI occurs as result of rapid acceleration/deceleration forces, causing deformation of brain tissue through shearing forces and stretching. Regions of varying densities stretch over each other, giving rise to widespread damage throughout the cerebral hemispheres, corpus callosum, and brain stem [16, 21]. Estimates suggest that DAI occurs in more than 80% of all motor vehicle induced TBI cases, and is consistently associated with worse outcome post-injury [2, 15, 16, 22]. Indeed, it has been reported that 58% of patients who had sustained a TBI and died within subsequent months demonstrated DAI [22]. Due to its nature, DAI is typically only detectable microscopically in post-mortem tissue, unless severe injury results in macroscopic white matter tears [16, 21]. As a result, diagnosing DAI in patients with conventional imaging techniques is difficult, and thus, the incidence of DAI may be under-diagnosed in TBI cases [16].

DAI was initially thought to be exclusively a primary injury event, a result of primary axotomy upon impact. However, recent studies have led to the understanding that DAI is in fact a progressive insult that leads to and prolongs neurological damage [15, 23, 24]. Axons are typically not completely torn upon the primary impact, but rather stretched, causing a localised intra-axonal change to the cytoskeleton. It is not until the secondary injury cascade commences that existing cytoskeletal damage causes disruption to the anterograde axoplasmic transport leading to axonal swelling with subsequent axonal disconnection [23, 25]. Furthermore, this cytoskeletal damage can disrupt sodium channel action, resulting in an influx of sodium and subsequent damage to voltage-gated calcium channels, causing a deleterious calcium influx. This in turn instigates the production of phospholipases and proteases, like calpains, damaging mitochondria and resulting in complete axonal separation and subsequent cell death [16, 25]. Undoubtedly, the consequences of DAI are often serious, as it is the most common cause of vegetative state and coma following TBI [16, 21, 26]. Many remain comatose, while survivors often have a poor quality of life due to considerable disability and impairment.

It is important to note that APP is frequently used as a highly sensitive maker of axonal injury [3, 4]. APP is typically transported via fast axonal transport in an anterograde direction, although a fraction of APP can travel retrogradely [27, 28]. Damage and stretching of axons through injury results in localised intra-axonal changes to the axolemma, disrupting fast axonal transport and facilitating accumulation of APP, which can be observed as early as 30 minutes following trauma [11]. Consequently, APP accumulations or swellings can be observed at the site of damage, and are easily detectable via immunohistochemistry [3, 4]. Whilst in this context APP can be used as a marker for injury, this is not its primary role in TBI.

## The Amyloid Precursor Protein

APP is best known and studied for its role as the source of the Amyloid-β (Aβ) peptide. The deposition of Aβ plaques is believed to play an important role in the pathogenesis of Alzheimer’s disease (AD), and accordingly, a vast amount of literature exists about the pathological roles of APP and its proteolytic products. However, the normal biological functions and actions of APP are yet to be clearly defined and understood.

APP is a constitutively expressed, highly conserved type-1 transmembrane glycoprotein. APP isoforms can be found in a number of places throughout the body including the spleen, thymus, kidney, lungs, liver, brain, heart, and platelets (reviewed in [29]), suggesting diverse physiological roles. Whilst APP is expressed in all cells that undergo cell-to-cell interactions, its expression is highest in neuronal cells and glia, particularly within the central nervous system (CNS). Here, APP serves a synaptic function [30], and is increasingly expressed in brain regions that undergo greater levels of synaptic modification, with expression of APP mRNA far greater in the foetal and developing brain than in the adult brain [31]. In neurons, APP is localised not only to somatodendritic and axonal compartments [27], but also to the presynaptic active zone [32].

### APP Isoforms & Structure

**Figure 1.**
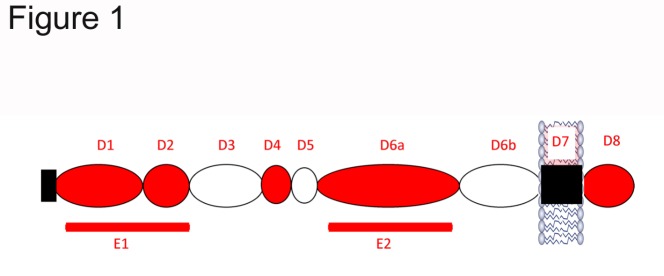
Representation of the structure of APP, highlighting its extracellular, transmembrane and intracellular domains.

As a single membrane-spanning protein of typically between 695 to 770 amino acids in length, APP comprises of a long extracellular N-terminal domain or ectodomain, a transmembrane region and a short intracellular C-terminal domain [33] (see Figure 1). The gene for APP is located on chromosome 21, and consists of 19 exons. Exons 7, 8 and 15 can be alternatively spliced to produce APP isoforms of which APP695, APP751 and APP770, defined by the number of amino acids they contain, are the most commonly studied [34]. Exon 7 codes for a Kunitz-type protease inhibitor (KPI) domain, found in only the APP751 and APP770 isoforms. In contrast, APP695 lacks the KPI domain [30, 34]. In addition, APP770 contains an OX-2 related domain [34]. Alternate splicing involving exon 15 typically occurs in leukocytes, and in the central nervous system in activated microglia and astrocytes, forming the leukocyte derived L-APP. Here, exon 15 is missing and exons 14 and 16 subsequently fuse together [30]. Whilst APP695 is found almost exclusively in neurons, APP751 and APP770 are found more extensively throughout other organs. However, they are expressed within glial cells, although at a considerably lower concentration than APP695 [35, 36].

Structurally, the APP extracellular domain comprises of up to six different sub-domains, depending on its isoform. These include the growth-factor like domain (D1), the copper binding domain (D2), an acidic domain (D3), a KPI and OX-2 domain (for isoforms APP751 and APP770 only) and a carbohydrate domain (D6) [37]. The combination of the D1 and D2 domains can also be referred to as the E1 domain. The D6 carbohydrate domain can be further divided into an E2 domain (D6a) and a juxtamembrane domain (D6b). APP has been shown to bind a variety of ligands, including metals, such as copper, iron and zinc, to cell surface and secreted molecules, as well as to heparan [38, 39]. Of particular interest is the heparan binding domain of the growth-factor like domain, which as discussed later, may mediate the neuroprotective activity of APP in TBI.

### APP Synthesis & Transport

APP is synthesised and translated in the endoplasmic reticulum, prior to travelling to the Golgi complex [32, 40]. Here, APP matures through the constitutive secretory pathway, and undergoes a variety of post-translational modifications including tyrosine sulphation, O- and N-linked glycosylation and phosphorylation [32, 41, 42]. APP is concentrated in the Golgi complex, and from here, travels through the central vacuolar system *en route* to the plasma membrane [27, 41]. Once attached, it must be cleaved via α-, β- and γ-secretase enzymes to produce APP fragments before these fragments can be released. Alternatively, APP may be internalized at the plasma membrane via clathrin- and dynamin-dependent pathways, and is either recycled back to the plasma membrane to follow the secretory pathway, or targeted towards the endosomal/lysosomal pathway [27, 43]. Only a fraction of synthesised APP will reach the cell surface for secretion, with only a small percentage of this APP actually being released [43, 44].

Transport of APP within neurons in the central nervous system varies slightly to that of other systems. APP is axonally sorted to vesicular compartments, and using kinesin and microtubules for transport, travels via fast axonal transport to the presynapse [27, 28, 32]. Here, it is incorporated into the presynaptic membrane, specifically into the presynaptic active zone, and to a lesser extent to free synaptic vesicles, suggesting a role in the physiology of neurotransmitter release [32].

## APP Proteolytic Processing

**Figure 2.**
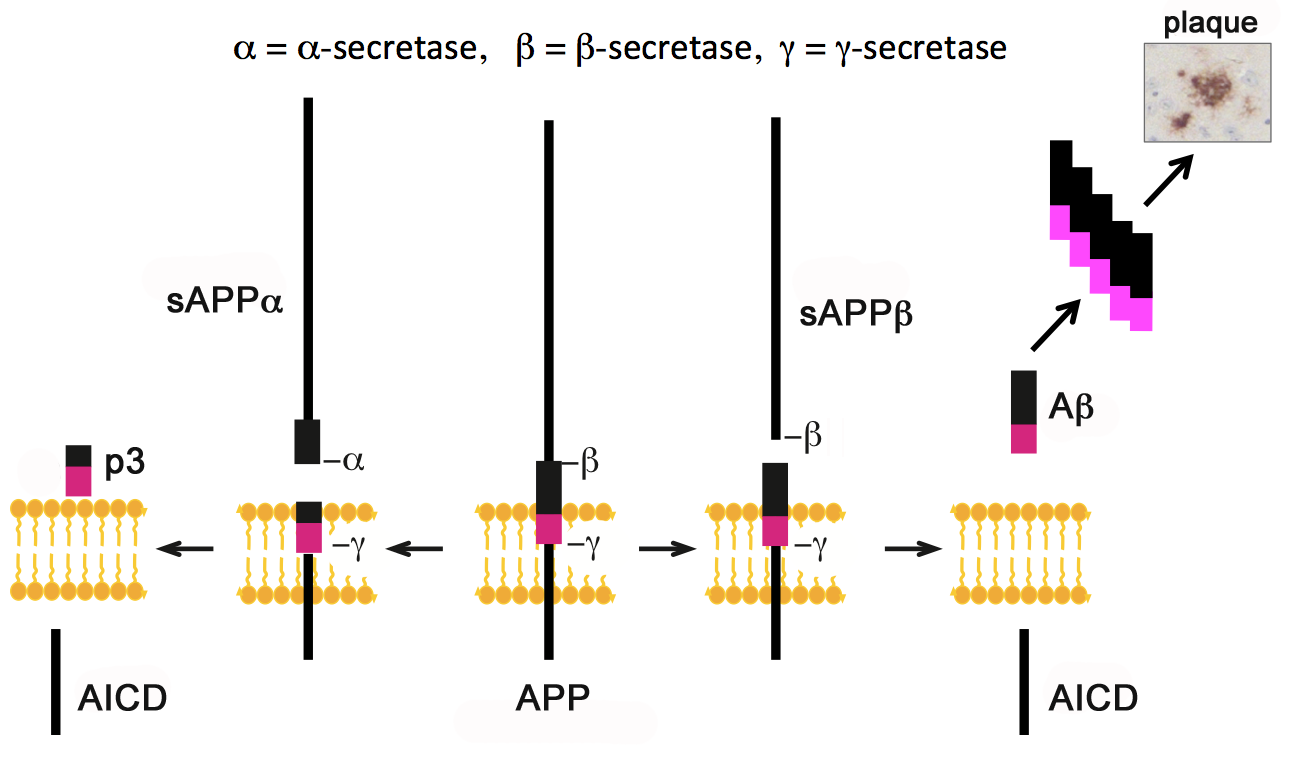
Representation summarising the major pathways of APP proteolytic processing via the α-, β- and γ-secretase enzymes.

Once mature, APP is able to undergo proteolytic cleavage on, or in close proximity, to the cell surface to produce smaller APP-derived metabolites [40]. Through cleavage, the integral transmembrane and C-terminal domains remain adhered to the cell membrane, with the extracellular domain released through a process referred to as ectodomain shedding [45, 46]. This cleavage process follows one of two major pathways, termed either the amyloidogenic or non-amyloidogenic pathway (see Figure 2).

### Amyloidogenic processing

Cleavage via the amyloidogenic pathway is a complex process involving cleavage of APP by the enzyme β-secretase BACE1 (Beta-site APP Cleaving Enzyme 1) to release the APP-β (sAPPβ) ectodomain and the 99 amino acid C-terminal membrane bound fragment C99. Further cleavage of C99 by the γ-secretase enzyme complex, that includes presenilin, results in the production of the neurotoxic amyloid-β (Aβ) peptide and the APP intracellular domain (AICD) (reviewed in [45]).

### Non-amyloidogenic processing

APP is preferentially cleaved via the non-amyloidogenic pathway, which occurs within the secretory pathway in the trans Golgi network and the cell surface [40]. Cleavage via α-secretase enzyme cleaves APP between amino acids 612 and 613 [44] producing the neuroprotective soluble APP-α (sAPPα) fragment and an 83 amino acid C-terminal fragment C83. Further cleavage via γ-secretase cleaves C83, producing the p3 fragment of unknown function, leaving the remaining AICD [45]. Importantly, cleavage via α-secretase cleaves APP in the middle of the region coding for Aβ, precluding Aβ formation [44, 47-49]. The majority of APP cleavage is via α-secretase, and not β- and γ-secretases as previously thought [43, 47, 48].

The enzymes responsible for endogenous α-secretase cleavage belong to the family of a disintegrin and metalloprotease (ADAM) enzymes. Specifically, the ADAM subtypes ADAM9, ADAM10 and ADAM17 have been shown to be the predominant α-secretase enzymes involved in APP cleavage [45, 49-51]. However, it appears that ADAM10 is the major α-secretase cleavage enzyme [45, 49], as even moderate neuronal overexpression was shown to strongly stimulate α-secretase cleavage of APP, delaying plaque formation, and alleviating cognitive defects in a transgenic AD mouse model [52, 53].

## The Functions of APP

Whilst the physiological function(s) of APP are yet to be fully understood, a variety of actions have been described and range from roles in metal homeostasis [54-57], binding and metabolism of proteoglycans [58-62], neuritogenesis [63], haemostasis and thrombosis [64, 65], glucose homeostasis [66], synaptogenesis and neuroprotection [67-70] and regulation of intracellular signalling [71, 72]. Many of these functions may account for the neuroprotective actions following brain trauma.

Knockout of APP leads to a range of deficits including reductions in body weight, grip strength, locomotor activity and brain weight, as well as age-related deficits in spatial learning [73]. Furthermore, histological analysis of the brain also revealed areas of gliosis, decreased neocortical and hippocampal levels of synaptophysin, and reduced dendritic lengths of hippocampal neurons with age [73, 74]. This supports a role for APP in a variety of cellular functions such as cell adhesion, neurite outgrowth and synaptogenesis.

APP belongs to a family of proteins that contains the APP homologues APP-like proteins, APLP1 and APLP2. The functions of these proteins greatly resemble APP, and differ mainly in their inability to produce Aβ plaques, the hallmark pathological feature of AD. It is believed that APLP1 and APLP2 may be able to take over the role of APP in APP knockout models [75, 76].

Particular focus has been placed on APP’s role in cell adhesion as a contact receptor, binding to other cells or to components of the extracellular matrix. This function is particularly important, as adhesion is known to regulate proliferation [77]. APP and its family members have been shown to form homo- and hetero-dimerisation complexes, associated with cell-cell adhesion promotion and subsequent *trans*-cellular adhesion *in vivo* [78].

In addition to its cell adhesion properties, APP has been proposed to play a role in neurotrophic and synaptotrophic functions such as neurogenesis, neurite outgrowth and synaptogenesis [79]. APP has been shown to promote functional synapse formation [63, 80], whilst reductions in APP expression have been associated with impaired neurite outgrowth and synaptic activity *in vivo* [81-83]. Indeed, APP’s metabolite sAPPα, has been shown to lead to neurite outgrowth like processes in cultured fibroblasts, cortical hippocampal neurons and in human neuroblastoma cells [84-90].

APP can also play a significant role in memory and cognitive function, most likely through being involved in processes like synaptogenesis, cell adhesion and neurite outgrowth. Knockout of APP in mice demonstrated age-related cognitive deficits including impairment in conditioned avoidance and Morris Water Maze tasks, highlighting a role for APP in processes that underlie learning and memory [74, 91, 92]. Furthermore, improvements in performing cognitive tasks were seen in an animal that had been exposed to an enriched environment, and was associated with a four-fold increase in APP protein levels, as well as an overall increase in the percentage of APP containing synapses in the hippocampus [93].

## Neuroprotective Actions of APP Derivatives

Whilst the proteolytic cleavage of APP produces a number of derivatives with varying pathological and physiological roles, it is the neuroprotective properties of these derivatives that are of particular importance in the context of brain trauma.

### sAPPβ

sAPPβ, the often forgotten peptide following β- and γ-secretase cleavage, has been considerably less studied than other metabolites like Aβ or sAPPα. sAPPβ shares the same extracellular sequence as sAPPα, with the exception of the final 16 amino acid sequence at the C-terminal. This sharing of the major domains results in sAPPβ and sAPPα having similar actions, including promotion of neurite and axonal outgrowth [94]. However, a number of differences have been observed. sAPPβ has been shown to offer almost 100 times less neuroprotective activity than sAPPα, particularly regarding protection from glucose deprivation and excitotoxicity [94, 95]. Furthermore, whilst sAPPβ has additionally been shown to help promote axonal elongation when added to cultured neurons, its effect is ten times lower than sAPPα [27, 96]. Additionally, key differences in sAPPβ appear to lie in its lack of activity in long-term potentiation (LTP) [96], as exogenous sAPPβ was less potent at restoring LTP levels to normal in rats [97]. Little is known about the role of sAPPβ in TBI, with one study showing sAPPβ levels were unchanged in the CSF of amateur boxers following a bout [98]. Given its reduced potency and efficacy in a variety of physiological settings, it is unlikely to confer the same level of neuroprotection in TBI as sAPPα. But this is a gap in the field that warrants further investigation to clarify sAPPβ‘s role in TBI.

### sAPPα

The APP metabolite with the greatest neuroprotective activity is sAPPα. *In vitro* studies have highlighted a number of functions of sAPPα (Table 1). sAPPα is able to enhance the long-term survival of cultured cortical neurons [99] and is believed to play a key role in the protection of cultured neuroblastoma cells against glutamate toxicity [100], as it can protect cultured neuronal cells from excitotoxic, metabolic and oxidative insults [29, 99, 101]. While these *in vitro* studies demonstrate the favourable properties of sAPPα, they often fail to take into account the heterogeneous nature of various ischaemic and traumatic insults, including those occurring in TBI, in both animals and humans. Accordingly, *in vivo* studies are typically more translatable to human conditions, assessing the dynamic nature of the diverse systems that interact during an injury.

*In vivo* studies have highlighted similar actions to those of *in vitro* studies (Table 2). sAPPα can enhance neurite outgrowth and promote cortical synaptogenesis [29, 99, 101]. Interestingly, sAPPα can also act synergistically with epidermal growth factor as a growth factor for neuronal progenitor cells in the subventricular zone of the lateral ventricle in adult mice [102]. This suggests a role for sAPPα in adult neurogenesis, as these cells retain the capacity to produce new neurons throughout adulthood.

**Table 1:** The neuroprotective and neurotrophic functions of sAPP *in vitro*

Model/Method	*In vitro* Neuroprotective & Neurotrophic Functions of sAPP	References
Cultured rat cortical neurons	Enhances long-term neuronal survival and neuronal extension	[84]
Cultured rat hippocampal and septal neurons & human cortical neurons	Protects against hypoglycaemic damage Reduces calcium ions; prevents calcium-mediated hypoglycaemia Protects against glutamate excitotoxicity	[67]
Application of Aβ to cultured rat hippocampal neurons	Reduces Aβ-induced injury Attenuates induction of reactive oxygen species Attenuates elevated intracellular calcium levels Protects against iron-induced oxidative injury	[149]
Cultured embryonic rat hippocampal neurons	Suppresses NMDA-induced currents	[99]
Cultured mouse epidermal growth factor responsive neurospheres	Regulates progenitor proliferation in the subventricular zone of lateral ventricle	[102]
Cultured mouse and rat hippocampal neurons	Regulates function of full-length APP in neurite outgrowth	[141]

## APP Has a Neuroprotective Role In TBI

APP has a long and significant association with TBI. As previously discussed, APP is acutely upregulated in injured neurons and astrocytes following TBI. Increases in APP protein levels within neuronal cell bodies and reactive astrocytes have been observed following experimental TBI including in rats [6, 103], pigs [104] and sheep [5], with similar findings in humans [3]. Increases in APP mRNA expression were seen as early as 30 minutes in the cerebral hemispheres, cerebellum and brainstem following diffuse TBI in sheep [5]. APP mRNA expression is regulated by many genes and proteins that are acutely increased following TBI including heat shock proteins and immediate early genes such as c-fos and c-jun [105].

**Table 2:** The neuroprotective and neurotrophic functions of sAPP *in vivo*

Model/Method	*In vivo* Neuroprotective & Neurotrophic Functions of sAPPα	References
**TBI models**		
Impact-acceleration model of diffuse TBI in rats	Improves motor outcome and attenuates axonal injury and neuronal cell loss	[124]
Controlled cortical impact (focal) TBI in mice followed by intracerebroventricular infusion	Improves motor and cognitive outcome	[152]
	Significantly improves cortical and hippocampal injury	
Controlled cortical impact (focal) TBI in APP-/- mice followed by intracerebroventricular infusion	Improves functional outcome, and reduces cortical and hippocampal cell damage	[131]
	Rescues deficits in APP-/- mice to be no longer significantly different to APP+/+ mice	
Weight-drop mechanical percussion model in mice	Etazolate, an α-secretase activator, reduces inflammation and cerebral oedema, improves memory and motor outcome and protects tissue	[132]
**Other injury models (non-TBI)**		
Four-vessel occlusion model of transient ischaemia in rat hippocampal neurons	Protects against transient cerebral ischaemic brain injury	[68]
Lateral ventricle infusion in rats	Increases synaptic density and memory retention; promotes synaptogenesis	[150]
Intracerebroventricular infusion	Enhances short- and long-term memory performance	[151]
	Blocks learning deficits induced by scopolamine	
Lateral ventricle infusion in mice	Increases number of epidermal growth factor responsive progenitors through increasing proliferation	[102]
Bilateral intrahippocampal electrode and cannula recordings & intrahippocampal infusion	Facilitates a role in LTP induction processes in rat dentate gyrus with effect isolated to sAPPα domain of APP	[97]
	Inhibition of α-secretase reduces LTP whilst exogenous sAPPα rescues it	
	Endogenous sAPPα is a key contributor to synaptic plasticity and spatial memory	
Transgenic mouse model with bovine ADAM10 over-expression	α-secretase over-expression shows neurotrophic effect of cortical cholinergic, glutamatergic and GABAergic presynaptic bouton populations	[101]

Initially, the purpose of the acutely increased APP expression after TBI was unclear. Due to the formation of the neurotoxic Aβ from APP cleavage, it had often been suggested that increased APP expression following TBI was detrimental, and would increase the risk of deposition of these Aβ plaques with the subsequent development of AD [106-108], particularly in susceptible individuals with the APOE ε4 allele [109, 110]. While this may represent a long term and unintended consequence of the upregulation of APP in response to acute brain injury, this theory has not been conclusively proven, with studies producing contradictory results. Whilst epidemiological reports have suggested there is a positive association between TBI and AD [108, 109, 111], other studies have found that TBI may not be a risk factor for the later development of AD [112, 113]. Similarly while some histopathological studies of individuals who died after suffering a single severe TBI demonstrate widespread Aβ deposition irrespective of age [114-116], others have concluded that Aβ deposition in victims below the age of 60 is a rare occurrence [117, 118]. Furthermore, the presence of Aβ plaques after TBI appears to decreases over time, with this attributed to an increase in the levels of the Aβ degrading enzyme, neprilysin [119]. This correlates with experimental studies employing transgenic mice with mutations which enable the development of AD-like pathology, which have not found that TBI accelerates Aβ deposition [120-122], unless a repetitive model of injury was used [123].

In contrast, Van den Heuvel and colleagues were the first to suggest that the upregulation of APP following TBI was actually beneficial and not detrimental, as increases in APP corresponded with increased preservation of neurons [5]. This not only identified that APP mRNA was a potential sensitive early indicator of neuronal injury, but importantly, that upregulation of APP serves as an adaptive and protective response to injury [5]. This hypothesis was supported, in part by our study by Corrigan and colleagues, reaffirming that endogenous APP serves a beneficial role following TBI [7]. Here, a lack of APP, through studying TBI in APP knockout mice (APP-/-), rendered the APP-/- mice more vulnerable to injury following a mild diffuse TBI. APP-/- mice demonstrated greater motor and cognitive deficits, increased vulnerability of neurons to injury and a defective reparative response to injury, compared to wildtype mice [7]. This was thought to be attributable to a lack of the metabolite sAPPα.

sAPPα is the metabolite that is believed to mediate the neuroprotective activity of APP, due to its previously described neuroprotective and neurotrophic actions in distinct injury models [67, 68]. Thornton and colleagues were the first to examine the neuroprotective role of exogenous sAPPα *in vivo* TBI [124]. sAPPα was administered via intracerebroventricular (ICV) infusion at 30 minutes following diffuse impact-acceleration TBI in rats, a clinically relevant model of TBI that produces DAI to mimic that seen in humans [125]. Rats treated with sAPPα after injury showed significant improvements in motor outcome over the seven day testing period when assessed on the rotarod when compared to vehicle control rats, and had reached baseline levels by day four post-injury [124]. Importantly, sAPPα was able to profoundly reduce the amount of axonal injury, and therefore injury severity, in the corpus callosum on day one following injury, reaching significance at days three and seven post-injury. This suggested that sAPPα may be efficacious at reducing the as-yet untreatable DAI. Furthermore, sAPPα administration was able to protect hippocampal neurons, significantly reducing the number of caspase-3 apoptotic cells in the hippocampus to a level that was only slightly more than non-injured [124]. Similar findings were observed following transient global ischaemia in rats, where post-traumatic ICV administration of sAPPα protected hippocampal neurons against ischaemic injury, as determined by the increased in neuronal survival and associated preservation of neuronal function [68].

Several mechanisms have been postulated as to how sAPPα exerts its neuroprotective effects. sAPPα can activate high conductance potassium channels, leading to hyperpolarisation of the cell and the suppression of calcium entry though voltage-dependent channels and NMDA receptors [95, 99, 126]. This will have a protective effect as excess calcium influx, which can commonly occur following excitotoxicity, can activate a number of destructive enzymes such as proteases and DNAses, which initiate cytoskeletal collapse. This may be a factor in the reduction of axonal injury mediated by sAPPα following TBI, as calcium induced activation of calpain pathways are capable of degrading the cytoskeletal network within the axon. Calpain-mediated degradation of the cytoskeleton has been shown to occur at sites of axonal damage and disconnection in numerous immunohistochemical studies employing antibodies directed towards its specific proteolytic breakdown products [127, 128]. Other mechanisms via which sAPPα may be neuroprotective include the ability to activate the transcription factor nuclear factor kappa B (NFκB) [129], which is important in promoting neuronal survival. This occurs by suppressing the expression of pro-apoptotic genes whilst upregulating anti-apoptotic genes [130].

Confirmation that sAPPα is the primary mediator of the neuroprotective activity of APP following TBI was that its administration restored deficits seen in APP-/- mice following trauma [131]. APP-/- mice demonstrated significantly poorer motor and cognitive outcomes following TBI when compared to wildtype mice, as assessed on the ledged beam and Barnes Maze, respectively. Following treatment with sAPPα, APP-/- mice performed no differently to wildtype mice on these assessments [131]. sAPPα treated APP-/- mice also demonstrated significant reductions in both cortical and hippocampal cell damage at both 24 hours and 7 days following trauma compared to untreated APP-/- mice, again resembling levels of wildtype mice in all instances [131]. These results, taken together with our earlier study in rats [124], highlight the neuroprotective effect of sAPPα following trauma.

Endogenous sAPPα levels can also be altered through pharmacological modulation of APP metabolism by using the α-secretase activator, Etazolate [132]. Etazolate treatment increased production of sAPPα, and was able to attenuate IL-1-mediated inflammation following TBI, including microglial activation, with a resultant improvement in cerebral oedema formation. This led to lasting memory improvements and motor performance due to the protection of cerebral tissue though the increase in sAPPα levels [132]. However, since α-secretase cleaves a number of other substrates besides APP, these effects by Etazolate cannot be unequivocally attributed to APP.

The modulation of other APP secretases has been recently applied as a therapeutic approach for TBI. Loane and colleagues used the inhibition of β- and γ- secretases to attenuate motor and cognitive deficits and reduce cell loss in mice following TBI [133]. This could be partially attributed to increases in sAPPα levels, as prevention of amyloidogenic processing could lead to increases in processing via the non-amyloidogenic pathway. Nonetheless, the aforementioned studies all indicate the potential of APP and sAPPα as a potential therapeutic agent.

## The Neuroprotective Active Site of APP in TBI

The specific regions within sAPPα that are responsible for conferring its neuroprotective activity have been identified. Assessment of both motor and cognitive outcome demonstrated that the D1 and D6a but not D2 domains (Figure 1) were equally as effective as full-length sAPPα [134]. Furthermore, administration of the D1 and D6a domains to rats post-TBI was able to significantly reduce axonal injury in the corpus callosum similar to that seen with sAPPα treatment, when compared to vehicle control and D2 treated rats [134].

Given the efficacy of the D1 and D6a domains, but not D2, it was hypothesised that a common functional site may exist governing this neuroprotective activity. It was postulated that a likely common functional feature is heparan binding, which is evident within both domains [94, 135]. The D1 domain has high structural similarity to growth-factor like domains, and displays strong affinity to heparan, particularly to heparan sulfate proteoglycans (HSPGs) [136-138]. Indeed, heparanases are able to prevent sAPPα from protecting cultured cells against glutamate toxicity and glucose-deprivation-induced injury [95].

To explore this hypothesis, treatment with a peptide encompassing the heparan binding domain in D1, namely APP residues 96-110 was investigated. ICV injection following trauma demonstrated continued efficacy in both APP-/- mice and in rats following diffuse TBI [139]. In APP-/- mice, APP96-110 was able to restore motor and cognitive deficits, associated with greater preservation of cortical and hippocampal tissue, so that they were no longer significantly different to APP wildtypes [139]. In diffuse TBI, APP96-110 was shown to improve motor and cognitive abilities of injured rats, and significantly reduced axonal injury in the corpus callosum at seven days post-injury [139]. Importantly, APP96-110 showed no difference in efficacy to the intact D1 protein indicating it was as fully active as D1 and sAPPα.

The efficacy of APP96-110 was related to APP’s heparan binding ability, since an APP96-110 analogue with reduced heparan binding, made by mutating the proposed heparan binding residues, had no neuroprotective effect [139]. This established that the neuroprotective activity of APP96-110 correlated to its ability to bind heparan.

## Proposed Mechanisms of Action

APP96-110 is able to bind to cell-surface or extracellular matrix bound HSPGs to elicit a neuritogeneic response [135, 137, 140]. The APP96-110 region contains a β hairpin loop formed by a disulphide bond between cysteines 98 and 105 [138, 140]. The presence of this bond has been shown to be critical for promoting neurite outgrowth [141] and the activation of MAP kinase [142]. Indeed, binding of this region to HSPGs can promote neurite outgrowth from central and peripheral neurons [140, 143, 144]. Furthermore, an antibody that binds to this region inhibits functional synapse formation [80], completely abolishes depolarisation induced neurite outgrowth [145].

HSPGs can act as either receptors or co-receptors [146], and the binding of sAPPα, through APP96-110, is proposed to lead to key physiological changes such as the regulation of cell adhesion, synaptogenesis, cell signalling and neurite outgrowth [135, 138, 140]. These changes are all important steps in promoting neuroplasticity and subsequent neurogenesis following TBI. Since HSPGs such as glypican and perlecan can inhibit the ability of APP to stimulate neurite outgrowth [144], there may be an interplay between APP and different HSPGs for APP96-110 to mediate its neuroprotective effects. As such, HSPGs may not be the target receptors for sAPPα or APP96-110 alone, rather HSPGs could bind to APP96-110 and aid in the binding of APP96-110 to its true neuroprotective receptor. Accordingly, identification of the definitive APP neuroprotective ligand remains an important goal to resolve the mechanism of action of sAPP in TBI.

## Development of Potential Therapeutics for TBI

Until this point, research has focused solely on achieving neuroprotective effects of exogenous APP molecules following ICV administration after TBI. However, the clinical application of a TBI therapy would ideally be via intravenous (IV), rather than ICV administration. This would facilitate an earlier administration by paramedics rather than requiring transport to a trauma unit. A challenge for many IV drugs that target the brain is the ability to penetrate the BBB. Following trauma, ensuing damage to the BBB facilitates a localised increase in permeability of blood contents into the brain parenchyma as early as 15 minutes after injury, lasting for up to four to six hours for large molecules. Permeability for smaller molecules, however, can last up to three to four days [147, 148]. Whilst this permeability does contribute to the injury process, it also provides a window of opportunity through which therapeutics may gain easier entry to the brain they otherwise may not [148].

An additional therapeutic challenge that often slows bench to bedside progress for TBI is that much experimental research focuses on an immediate time point after injury, generally up to an hour post-TBI. As the time frame between injury and the medical diagnosis of trauma in human situations may often far exceed one hour, an ideal therapy for TBI should demonstrate efficacy up to several hours post injury. As such, research focusing on more clinically relevant time frames would help overcome this therapeutic challenge.

## Conclusion

This review has focused on the neuroprotective actions conferred by APP following TBI. Acute upregulation of APP has been shown to serve a protective, rather than detrimental role following trauma. This is now believed to be due to the presence of sAPPα, a metabolite that may bind to HSPGs or another receptor via its heparan binding sites, in particular, amino acid residues 96-110. A peptide encompassing APP96-110 has been shown to offer potent neuroprotective activity following TBI, including improved motor and cognitive outcome and reduced tissue loss. Most importantly, treatment with APP96-110 reduced axonal injury and overall injury severity. Accordingly, further development of APP96-110 as a therapeutic for TBI, and particularly DAI which currently lacks an efficacious treatment, is warranted as its efficacy at improving functional outcome and reducing injury severity is significant. Therefore, sAPPα/APP96-110 present as a novel and viable treatment offering substantial neuroprotective and neurotrophic effects for ameliorating acute brain injury.
